# Genetic assessment of productive and reproductive traits in Friesian, native, and crossbred cattle in Egypt

**DOI:** 10.1007/s11250-024-04153-1

**Published:** 2024-10-15

**Authors:** Abdullah Ali Ghazy, Abdelhamid Saeed Abo El-Enin, Anas Abd El-Salam Abou El-Anine Badr, Hassan Ghazy El-Awady, Ibrahim Atta Mohammed Abu El-Naser

**Affiliations:** 1 Faculty of Agriculture, Department of Animal Reproduction, Suez Canal University, Ismailia, 41522 Egypt; 2 Ministry of Agriculture, Animal Production Research Institute, Dokki, Egypt; 3 Faculty of Agriculture, Animal Production Department, Kafrelsheikh University, Kafrelsheikh, 33516 Egypt; 4 Faculty of Agriculture, Department of Animal, Poultry and Fish Production, Damietta University, Damietta, Egypt

**Keywords:** Genetic parameters, Phenotypic correlations, Breeding values, Friesian, Native, Crossbred cows

## Abstract

This study utilized a dataset comprising 3023 lactation records for Friesian cows, 596 records for Native cows (Baladi), and 1189 records for Crossbred cows spanning from 1994 to 2015. The objective was to estimate and assess genetic and phenotypic parameters and breeding values for 305-day milk yield (305-DMY), lactation period (LP), calving interval (CI), and days open (DO) within the Egyptian dairy context. The motivation for this research stemmed from the need to understand the genetic potential of different cattle genotypes in Egypt and identify opportunities for enhancing dairy production. Data were analyzed using the linear mixed model least squares and maximum likelihood (LSMLMW) and multiple-trait derivative-free restricted maximum likelihood (MTDFREML) programs. The analytical model included fixed effects such as season and year of calving, parity, and genotype groups, while random effects included animal and error. Unadjusted means for 305-DMY, LP, CI, and DO were calculated for each genotype group. Genotype groups significantly impacted all studied traits. Heritability estimates varied across genotype groups, with higher estimates observed in Crossbred (0.32, 0.26, 0.25, 0.23) and Native cows (0.26, 0.28, 0.28) compared to Friesian cows (0.24, 0.22, 0.16, 0.17) for productive and reproductive traits, respectively. Genetic correlations among traits ranged from 0.10 to 0.86 for the three genotype groups, while corresponding phenotypic correlations were generally small to moderate and positive. Regarding breeding values, the accuracy estimates suggested that both sires and cows could contribute to genetic improvement. This indicates the potential for enhancing dairy production through selective breeding strategies.

## Introduction

In Egypt, the dairy industry represents a significant portion, accounting for 30–40% of total investments in the animal production sector. Native Egyptian cows play a crucial role in the dairy industry, known for their adaptation to local environmental conditions and their tolerance to common diseases. However, their population is declining, which poses a threat to their genetic diversity and resilience (Abo-Elenin 2018; Sanad and Hassanane 2019).

Regarding the distribution of the cow population in Egypt, data from CAPMAS (2019) indicates that the total number of cows in Egypt is reported as 2,808,640. Within this total, foreign cows comprise approximately 7.7% of the population, totaling 215,887, while Baladi and crossbred cows together represent approximately 92.3%, accounting for 2,592,753 individuals. Most of the purebred Friesian cows are found in large private farms due to their specialized nutritional and management requirements. The increasing number of crossbred Friesian cows has led to a decline in the population of native cows, with the Damietta cows being one of the most prominent native breeds (Salah Galal 2007).

In recent decades, Egyptian farmers have increasingly adopted agricultural mechanization and have replaced local livestock with crossbred Friesian breeds. This crossbreeding, characterized by crossbreeding with local cows, is more productive in terms of milk production and resistant to common diseases compared to pure Friesian breeds, in addition to being more cost-effective.

The productive characteristics of Friesian cattle in Egypt, as well as native cows and their crosses, have been extensively studied, and genetic parameters for productive traits of imported cattle and their crosses with native cows have been estimated (Mostageer et al. 1990). Enhancing production efficiency can be achieved through improved environmental conditions, enhancing the mean breeding values of the population’s members, or a combination of both (Katkasame et al. 1996). Several researchers have investigated genetic and non-genetic factors affecting productive and reproductive traits in Friesian, Native, and their Crossbred cows in Egypt, including studies by Abo-Elenin (2018), Awady et al. (2016), El-Awady (2013), Hussein et al. (2016), and Sanad and Gharib (2021).

The objectives of this study are to estimate genetic and phenotypic parameters and breeding values for 305-day milk yield (305-DMY), lactation period (LP), calving interval (CI), and days open (DO), and to assess these productive and reproductive traits for Friesian, Native, and Crossbred cows under Egyptian conditions.

## Materials and methods

### Data

This study was conducted with approval from the Research Ethics Committee of the Faculty of Agriculture at Suez Canal University (reference number 42/2023). The dataset used in the analysis comprised 3023 normal lactation records of Friesian cows (703 cows and 74 bulls), 596 normal lactation records of Native cows (212 cows and 36 bulls), and 1189 normal lactation records of Crossbred cows (316 cows and 51 bulls). These records span from 1994 to 2015 and were sourced from Sakha and El-Karada farms in Kafr-El-Sheikh governorate (for Friesian cows), El-Serow farm in Damietta governorate (for Native cows), and El-Gemeza farm in EL-Gharbia governorate (for Crossbred cows). All three farms are affiliated with the Animal Production Research Institute (APRI), Agriculture Research Center, Ministry of Agriculture, Dokki, Giza, Egypt.

The animals were loosely housed in open shed systems and were subjected to similar systems of feeding and management across all farms, as described by Abo-Elenin (2018). The traits investigated included 305-day milk yield (305-DMY, kg), lactation period (LP, days), calving interval (CI, days), and days open (DO, days).

### Data analysis

Data were analyzed using the LSMLMW computer program (Harvey 1990) for obtained the main effects. The mixed model used was:

$$\text{Y}_{\text{ijklmn}}=\mu+\text{S}_\text{i}+\text{SE}_\text{j}+\text{Y}_\text{k}+\text{P}_\text{l}+\text{GE}_\text{m}+\text{e}_\text{ijklmn}$$

Where: $\:Yijklmn$ = individual observation; µ = overall means; Si = random effect of sire, SEj = fixed effect of season of calving (autom, spring, summer and winter) (j = 1 to 4); Y_k_ = fixed effect of year of calving (k = 1994 to 2015); P_l_ = fixed effect of parity (l = 1 to 6), GE_m_ = fixed effect of the genotype group (m = Friesian, Native and Crosses) and e_ijklmn_ = the residual effect.

Secondly, data analyzed to estimate the variance and covariance component with derivative-free restricted a maximum likelihood (REML) procedure using the MTDFREML program of (Boldman et al. 1995). The assumed model was:

$$\:Y=\text{Xb}+\text{Zu}+W\text{pe}+e$$

Where: Y = a vector of observations, b = a vector of fixed effects with an incidence matrix X, u = a vector of random animal effects with an incidence matrix Z, p_e_ = a vector of permanent environmental effect with an incidence matrix W, e = a vector of residual effect. To estimate heritability (h^2^_a_) the following equation was used:

$$\text{h}^{2}_\text{a}=\sigma^{2}_\text{a}\:/\:(\sigma^{2}_\text{a}+\sigma^{2}_\text{pe}+\sigma^{2}_\text{e})$$

Where: σ^2^_a_ = additive genetic variance; σ^2^_pe_ = permanent environmental variance and σ^2^_e_ = the random residual effect associated with each observation.

Genetic correlations (rg) between any two traits were estimated according to Legates and (Legates and Warwick 1990), while phenotypic correlations (rp) were calculated according to (Turner and Young 1969).

Cow breeding values (CBV) were evaluated using their own records, while dam and sire breeding values were evaluated without their own records. The MTDFREML program was used to estimate the best linear unbiased predictions (BLUP) of calculated breeding values (BV’s) for all animals’ pedigree files for multi-traits analysis.

## Results

### Descriptive statistics

Unadjusted means, standard deviations, and coefficients of variation (CV %) for the various traits are presented in Table 1. The coefficients of variation indicate variability within each genotype group. In Friesian cows, the coefficients ranged from 22.92 to 59.43%, in Native cows from 22.00 to 64.75%, and in Crosses cows from 24.74 to 44.64%. These variations suggest differing levels of trait expression and potential for genetic improvement across the genotype groups.

Additionally, the influence of the year of calving and genotype groups on key traits such as 305-day milk yield (305-dMY), lactation period (LP), calving interval (CI), and days open (DO) was significant (*P* < 0.01). The season of calving was also found to have a highly significant effect on 305-dMY (*P* < 0.01), and a significant effect on LP (*P* < 0.05), although no significant effect was observed on CI and DO. Parity showed a highly significant effect on all studied traits except LP.

**Table 1:** Phenotypic means, standard deviations (SD), coefficient variability (C.V%), significance levels of independent variables, and distributions of the data of 305-DMY, LP, CI, and DO in Friesian (F), native (N), and crossbred (FN) cows in Egypt

Genotype Group	Traits	305-dMY, kg	LP, d	CI, d	DO, d
Friesian (F)	**Mean ± SD**	3597 ± 1421.6	362.1 ± 117.5	524 ± 120.1	198.3 ± 117.8
	**CV%**	39.52	32.45	22.92	59.43
Native (N)	**Mean ± SD**	1399 ± 906.0	199.0 ± 70.7	499 ± 109.7	168.8 ± 99.5
	**CV%**	64.75	35.54	22.00	58.96
F x N (FN)	**Mean ± SD**	2672 ± 1178.6	394.6 ± 118.8	556 ± 137.6	215.3 ± 96.1
	**CV%**	44.12	30.10	24.74	44.64
Variable	**Levels of significance (F)**				
	**Traits**				
	**305-DMY**	**LP**	**CI**	**DO**	
Season of calving	5.94**	3.52*	1.35^ns^	1.32^ns^	
Year of calving	40.38**	3.30**	3.59**	3.48**	
Parity	91.30**	3.19*	15.49**	17.40**	
Genotype group	272.78**	185.09**	14.52**	20.27**	

ns = not significant, * significant at *P* < 0.05 and ** significant at *P* < 0.01

### Genetic parameter assessments

Genetic parameter assessments are vital for decision-making processes related to selection methods, prediction of responses to selection, choice of breeding systems, and evaluation of genetic gains. Table 2 presents estimates of (co)variance components for various traits in Friesian, Native, and Cross cows.

The current estimates of additive genetic variance (σ^2^_a_) for all traits were found to be lower than the residual variance (σ^2^_e_). Notably, in fertility-related traits, a significant proportion of the total variation was attributed to residual variance effects. This observation underscores the potential for achieving reasonable rates of improvement in these traits through effective management and environmental conditions.

Direct heritability estimates for 305-DMY (305-day milk yield) and LP (lactation period) in Friesian cows were moderate, at 0.24 and 0.22, respectively. These values were lower than those observed in Native cows (0.26 for 305-DMY and 0.28 for LP) and Crosses cows (0.32 for 305-DMY and 0.26 for LP). Conversely, CI (calving interval) and DO (days open) in Friesian cows exhibited the lowest heritability values, measuring 0.16 for CI and 0.17 for DO, in comparison to Native cows (0.24 for CI and 0.27 for DO) and Crosses cows (0.25 for CI and 0.23 for DO), as detailed in Table 2.

**Table 2:** Estimates of variance components and genetic parameters for studied traits through animal model for Friesian (F), native (N) and crosses (FxN) cows in Egypt

Genotype Group	Friesian Cows				Native Cows				Crosses Cows			
Traits	305-DMY	LP	CI	DO	305-DMY	LP	CI	DO	305-DMY	LP	CI	DO
σ^2^_a_	3.23	3.36	2.68	3.53	2.54	3.10	3.22	3.50	4.50	3.30	3.88	3.17
σ^2^_pe_	2.61	3.25	2.86	12.88	2.60	3.21	3.36	3.95	4.03	3.55	4.06	4.50
σ^2^_e_	7.92	8.72	11.33	4.15	4.72	4.69	6.74	5.47	5.73	5.72	7.34	5.92
σ^2^_p_	13.76	15.32	16.87	20.55	9.86	11.00	13.32	12.91	14.26	12.57	15.28	13.59
h^2^_a_	0.24	0.22	0.16	0.17	0.26	0.28	0.24	0.27	0.32	0.26	0.25	0.23
c^2^	0.19	0.21	0.17	0.63	0.26	0.29	0.25	0.31	0.28	0.28	0.27	0.33
e^2^	0.57	0.57	0.67	0.20	0.48	0.43	0.51	0.42	0.40	0.46	0.48	0.44

σ^2^_a_ = direct additive genetic variance, σ^2^_pe_ = maternal permanent environmental variance, σ^2^_e_ = residual (temporary environmental variance), σ^2^_p_ = phenotypic variance, h^2^_a_ = direct heritability, c^2^ = fraction of phenotypic variance due to maternal permanent environmental effects and e^2^ = fraction of phenotypic variance due to residual effects

### Genetic and phenotypic correlations

Genetic and phenotypic correlations, along with permanent and residual ratios among different studied traits for the three genotypes, are presented in Table 3. Genetic correlations between 305-DMY and LP, CI, and DO in Friesian cows were moderate and positive (0.45, 0.31, and 0.29), respectively, and were close to the corresponding correlations in Crosses cows (0.47, 0.32, and 0.30). In contrast, these correlations were lower in Native cows (0.35, 0.24, and 0.22), respectively. Additionally, genetic correlations among LP and CI, as well as LP and DO, were higher in Crosses cows (0.61 and 0.86) and Friesian cows (0.58 and 0.83) compared to Native cows (0.46 and 0.65). Genetic correlations between CI and DO were low for all three genotype groups (0.12, 0.10, and 0.12). The residual ratios among the studied traits ranged from 0.01 to 0.40 for Friesian cows, 0.009 to 0.31 for Native cows, and 0.01 to 0.41 for Crosses cows. In all three genotype groups, the highest ratio was observed between 305-DMY and DO, while the lowest ratio was between LP and DO.

**Table 3:** Estimation of genetic correlation (r_g_), phenotypic correlation (r_p_), permanent ratio (r_pe_) and residual ratio (r_e_) for 305-DMY, LP, CI, and DO of Friesian, native and crosses cows

	Trait 1	Trait 2	*r*_g_	*r*_*p*_	*r*_pe_	*r*_e_
Friesian Cows	305-DMY	LP	0.45	0.18	0.25	0.10
		CI	0.31	0.32	0.85	0.18
		DO	0.29	0.19	0.01	0.40
	LP	CI	0.58	0.06	0.13	0.17
		DO	0.83	0.22	0.01	0.01
	CI	DO	0.12	0.18	0.12	0.30
Native Cows	305-DMY	LP	0.35	0.14	0.20	0.08
		CI	0.24	0.25	0.67	0.14
		DO	0.22	0.14	0.01	0.31
	LP	CI	0.46	0.04	0.10	0.13
		DO	0.65	0.18	0.10	0.10
	CI	DO	0.10	0.14	0.095	0.23
Crosses Cows	305-DMY	LP	0.47	0.19	0.26	0.10
		CI	0.32	0.33	0.99	0.19
		DO	0.30	0.20	0.01	0.41
	LP	CI	0.61	0.06	0.13	0.18
		DO	0.86	0.23	0.01	0.01
	CI	DO	0.12	0.19	0.12	0.31

### Breeding values

The range of expected breeding values (EBVs) for cows (EBV’c) and sires (EBV’s) for Friesian, Native, and Crosses cows are presented in Table 4. The EBV’c range for Friesian cows was 1397 kg for 305-DMY, 31.93 days for LP, 13.84 days for CI, and 16.57 days for DO. For Native cows, the range was 641.3 kg for 305-DMY, 28.05 days for LP, 117.7 days for CI, and 101.86 days for DO. Crosses cows showed a range of 1258.4 kg for 305-DMY, 220.55 days for LP, 89.65 days for CI, and 84.37 days for DO. The EBV’s range for Friesian cows was 881 kg for 305-DMY, 16.17 days for LP, 7.84 days for CI, and 10.87 days for DO. For Native cows, the range was 345.4 kg for 305-DMY, 119.9 days for LP, 139.81 days for CI, and 99.88 days for DO. Crosses cows showed a range of 465.3 kg for 305-DMY, 121 days for LP, 155.98 days for CI, and 121.77 days for DO.

The range of expected breeding values for dams (EBV’d) was 1002.1 kg for 305-DMY, 25.14 days for LP, 11.83 days for CI, and 13.66 days for DO in Friesian cows; 283.8 kg, 99.00 days, 86.57 days, and 81.62 days for Native cows; and 347.6 kg, 25.30 days, 43.01 days, and 64.24 days for Crosses cows.

**Table 4:** Range of expected breeding values (EBV’’s) through cows, sires, dams and it’s percentage of accuracy’s (%) for 305-DMY, LP, CI and DO in Friesian, native and crosses cows

Genotype	Traits	Cows (EBV’c)				
		Min.	Max.	Range	SEP	Accuracy%
**Friesian**	**305-DMY**	-591.8	805.2	1397	71.5–112.2	00–84.7
	**LP**	-10.571	21.362	31.93	12.1–25.3	00–95.7
	**CI**	-5.148	8.69	13.84	19.8–20.9	00–89.1
	**DO**	-6.831	9.735	16.57	0.95–1.65	00–90.2
**Native**	**305-DMY**	-243.1	398.2	641.3	69.3–75.9	69.3–75.9
	**LP**	-20.35	7.7	28.05	67.1–75.9	70.4–75.9
	**CI**	-32.67	85.03	117.7	23.1–53.9	75.9–86.9
	**DO**	-41.36	60.5	101.86	31.9–33	64.9–71.5
**Crosses**	**305-DMY**	-232.1	1026.3	1258.4	62.7–92.4	22–31.9
	**LP**	-153.45	67.1	220.55	34.1–89.1	53.9–75.9
	**CI**	-27.28	62.37	89.65	23.1–30.8	74.8–89.1
	**DO**	-22.55	61.82	84.37	29.7–51.7	85.8–86.9
**Sires (EBV’s)**						
**Friesian**	**305-DMY**	-414.7	466.4	881.1	35.2–112.2	00–104.5
	**LP**	-7.7	8.47	16.17	9.9–25.3	00–106.7
	**CI**	-4.334	3.509	7.843	5.5–20.9	00–105.6
	**DO**	-4.433	6.435	10.87	0.45–1.65	00–105.6
**Native**	**305-DMY**	-135.3	210.1	345.4	17.6–26.4	52.8–75.9
	**LP**	-72.6	47.3	119.9	17.6–52.8	67.1–92.4
	**CI**	-65.34	74.47	139.81	45.1–50.6	39.6–94.6
	**DO**	-42.13	57.75	99.88	31.9–33	63.8–91.3
**Crosses**	**305-DMY**	-184.8	280.5	465.3	51.7–89.1	67.1–95.7
	**LP**	-72.6	48.4	121	17.6–34.1	30.8–95.7
	**CI**	-85.14	70.84	155.98	42.9–50.6	61.6–70.4
	**DO**	-74.14	47.63	121.77	40.7–48.4	40.7–63.8
**Dams (EBV’d)**						
**Friesian**	**305-DMY**	-457.60	544.50	1002.10	81.4–112.2	00–105.6
	**LP**	-11.61	13.53	25.14	15.4–25.3	00–86.9
	**CI**	-5.47	6.36	11.83	15.3–20.9	00–79.2
	**DO**	-6.95	6.71	13.66	1.11–1.65	00–81.4
**Native**	**305-DMY**	-165.00	118.80	283.80	118.8–133.1	34.1–40.7
	**LP**	-41.80	57.20	99.00	27.5–52.8	34.1–38.5
	**CI**	-39.38	47.19	86.57	22–42.9	90.2–91.3
	**DO**	-29.81	51.81	81.62	18.7–29.7	63.8–69.3
**Crosses**	**305-DMY**	-122.10	225.50	347.60	135.3–138.6	30.8–31.9
	**LP**	-12.10	13.20	25.30	73.7–75.9	24.2–69.3
	**CI**	-15.29	27.72	43.01	11–18.7	46.2–52.8
	**DO**	-29.59	34.65	64.24	16.5–27.5	57.2–62.7

## Discussion

### Descriptive statistics

The descriptive statistics presented in this study provide valuable insights into the variation of key traits across different genotype groups of dairy cows. The coefficients of variation (CV %) for traits such as milk yield, lactation period, calving interval, and days open demonstrate variability within and among Friesian, Native, and Crosses cows. These findings are consistent with prior research by Abo-Elenin (2018), who reported similar results for Friesian, Baladi, and Crosses cows in a different dataset.

Further insights into specific traits are provided by studies such as El–Awady and Abu El-Naser (2017) and El–Awady et al. (2017). For instance, El–Awady and Abu El-Naser (2017) reported overall means for lactation period (LP), days open (DO), and calving interval (CI) in Friesian cows, while El–Awady et al. (2017) provided mean values for 305-day milk yield (305-dMY), LP, CI, and DO. These findings contribute to our understanding of trait variation and provide a basis for comparison with the current study.

Moreover, the significant influence of the year of calving and genotype groups on key traits such as 305-dMY, LP, CI, and DO highlights the multifactorial nature of dairy cow performance. These results are consistent with prior research, including studies by El-Awady and Oudah (2012), Faid-Allah (2015), Abo-Elenin (2018), and Sanad and Gharib (2021), which investigated the effects of various factors on dairy cow traits in Egypt.

The observed significant effects of the year of calving, season of calving, and parity on studied traits underscore the importance of considering environmental and management factors in dairy herd management and breeding programs. These findings align with previous research and emphasize the need for targeted interventions to optimize dairy cow performance and reproductive efficiency under local conditions.

### Genetic parameter assessments

These findings regarding variances and heritabilities align with previous studies conducted by Awady et al. (2016), El–Awady et al. (2017), and Abo-Elnin (2018) on Friesian, Native, and crossbred cows in Egypt. The observed patterns of additive genetic variance and heritability across different traits and genotype groups provide valuable insights for future breeding strategies and population enhancement efforts.

Faid-Allah (2015) reported relatively lower heritability values for LP and DO in Holstein cows, suggesting potential challenges in improving these traits through selective breeding. Similarly, Hussein et al. (2016) found lower heritability estimates for milk yield and LP in Baladi cows, indicating limited genetic control over these traits in this breed.

The studies conducted by El–Awady et al. (2017), El–Awady and Abu El-Naser (2017), Sanad and Gharib (2021), and Abu El-Naser et al. (2020) further contribute to our understanding of the genetic components of various traits in Friesian and Native cows. These collective findings provide a comprehensive perspective on the heritability of important traits in different cattle breeds, which is essential for designing effective breeding and selection programs aimed at improving productivity and reproductive performance.

### Genetic and phenotypic correlations

Understanding the genetic correlations and residual ratios among various traits in different cattle genotypes is crucial for making informed decisions related to breeding strategies and genetic improvement. The observed moderate to positive genetic correlations between 305-DMY and LP, CI, and DO in Friesian and Crosses cows indicate a tendency for these traits to be genetically linked, suggesting that selection for higher milk yield may also lead to changes in lactation period, calving interval, and days open. However, the lower correlations observed in Native cows suggest a weaker genetic relationship between these traits in this genotype. The higher genetic correlations among LP and CI, as well as LP and DO, in Crosses and Friesian cows compared to Native cows imply a stronger genetic association between these reproductive traits in the former genotypes. This suggests that selection for improved lactation period may also have a more significant impact on calving interval and days open in Crosses and Friesian cows compared to Native cows. The low genetic correlations between CI and DO across all genotype groups suggest that these two reproductive traits may be governed by different genetic mechanisms and are less influenced by each other’s genetic effects. The observed residual ratios provide insights into the proportion of total phenotypic variation not explained by genetic or permanent environmental effects, representing the environmental variation unique to each individual. The higher residual ratios for certain traits in Native cows compared to Friesian and Crosses cows may indicate a greater influence of environmental factors on these traits in the Native genotype.

The observed genetic correlations between various production and reproductive traits provide valuable insights into the interrelationships among these traits in different cattle genotypes. The moderate to high positive correlations between 305-DMY and LP, CI, and DO indicate that selection for increased milk yield may lead to improvements in lactation period and reproductive performance, consistent with findings reported by Awady et al. (2016) in Friesian cows. The differences in genetic correlations among genotype groups highlight potential genotype-specific responses to selection for desired traits. For instance, the higher genetic correlations between LP and CI, as well as LP and DO, in Crosses and Friesian cows compared to Native cows, suggest that selection for prolonged lactation period may have a more pronounced effect on calving interval and days open in these genotypes. These findings are consistent with studies conducted by Hussein et al. (2016) on Baladi cows and Awady et al. (2016) on Friesian cows. The observed residual ratios among traits reflect the degree of non-genetic variation shared among them. The higher residual ratios between 305-DMY and DO suggest that environmental factors may play a larger role in determining days open compared to other traits. These findings are in line with the results reported by Faid-Allah (2015) and provide further evidence of the complex interplay between genetics and environmental factors in determining reproductive performance in dairy cows. Overall, the genetic and phenotypic correlations presented in this study contribute to our understanding of the relationships among important production and reproductive traits in different cattle genotypes, which is essential for designing effective breeding programs aimed at improving productivity and reproductive efficiency.

### Breeding values

The breeding values presented in this study provide valuable insights into the genetic potential and reliability of cows and sires for various production and reproductive traits in different cattle genotypes. These breeding values are essential for selection and improvement programs aimed at enhancing productivity and reproductive efficiency in dairy herds.

The observed range of breeding values for traits such as 305-DMY, LP, CI, and DO reflects the genetic variability present within and among different genotype groups. The higher range of breeding values observed in Crosses cows for traits like LP and CI suggests potential opportunities for genetic improvement in these traits through selective breeding. These findings are consistent with previous studies conducted by Abo-Elenin (2018), Awady et al. (2016), and Sanad and Gharib (2021).

The accuracy and reliability of breeding values are crucial for effective selection decisions. The insights provided by El–Awady and Abu El-Naser (2017) and Sanad and Gharib (2021) regarding the accuracy and reliability of breeding values for traits like LP, DO, and CI emphasize the utility of these values in guiding breeding programs for improved performance and productivity.

## Conclusion

The findings of this study underscore the robust genetic potential of Native Egyptian cows, complemented by Crosses cows’ ability to deliver commendable milk production, despite slightly extended calving intervals. These attributes align well with the Egyptian environmental conditions, making them suitable choices for local cattle farming.

Moreover, Egyptian Native cows exhibit unique characteristics, including heightened disease resistance and remarkable adaptability to the local environment. These traits emphasize the importance of preserving this indigenous breed for maintaining genetic diversity in the region’s cattle population.

Building upon these insights, we recommend a concerted effort to conserve and safeguard Egyptian Native cows. By preserving their unique genetic traits, we can not only bolster the resilience of local cattle farming but also explore their potential in enhancing the genetic traits of high-economic-value breeds, such as pure Friesian cows.

Through strategic conservation and targeted breeding programs, we can leverage the strengths of Native Egyptian cows to contribute to the sustainability and productivity of the dairy industry in Egypt. This approach will not only benefit local farmers but also promote the long-term viability of the region’s cattle farming sector.

## Data Availability

The study data can be obtained from the corresponding author upon making a reasonable request.
